# Hyperosmolarity Invokes Distinct Anti-Inflammatory Mechanisms in Pulmonary Epithelial Cells: Evidence from Signaling and Transcription Layers

**DOI:** 10.1371/journal.pone.0114129

**Published:** 2014-12-05

**Authors:** Franklin L. Wright, Fabia Gamboni, Ernest E. Moore, Trevor L. Nydam, Sanchayita Mitra, Christopher C. Silliman, Anirban Banerjee

**Affiliations:** 1 Department of Surgery/Trauma Research Center, University of Colorado Denver, Aurora, Colorado, United States of America; 2 Department of Surgery, Denver Health Medical Center, Denver, Colorado, United States of America; 3 Department of Pediatrics, University of Colorado Denver, Aurora, Colorado, United States of America; The Hong Kong Polytechnic University, Hong Kong

## Abstract

Hypertonic saline (HTS) has been used intravenously to reduce organ dysfunction following injury and as an inhaled therapy for cystic fibrosis lung disease. The role and mechanism of HTS inhibition was explored in the TNFα and IL-1β stimulation of pulmonary epithelial cells. Hyperosmolar (HOsm) media (400 mOsm) inhibited the production of select cytokines stimulated by TNFα and IL-1β at the level of mRNA translation, synthesis and release. In TNFα stimulated A549 cells, HOsm media inhibited I-κBα phosphorylation, NF-κB translocation into the nucleus and NF-κB nuclear binding. In IL-1β stimulated cells HOsm inhibited I-κBα phosphorylation without affecting NF-κB translocation or nuclear binding. Incubation in HOsm conditions inhibited both TNFα and IL-1β stimulated nuclear localization of interferon response factor 1 (IRF-1). Additional transcription factors such as AP-1, Erk-1/2, JNK and STAT-1 were unaffected by HOsm. HTS and sorbitol supplemented media produced comparable outcomes in all experiments, indicating that the effects of HTS were mediated by osmolarity, not by sodium. While not affecting MAPK modules discernibly in A549 cells, both HOsm conditions inhibit IRF-1 against TNFα or IL-1β, but inhibit p65 NF-kB translocation only against TNFα but not IL-1β. Thus, anti-inflammatory mechanisms of HTS/HOsm appear to disrupt cytokine signals at distinct intracellular steps.

## Introduction

Clinically, hypertonic saline (HTS) has been used to treat post-traumatic acute lung injury (ALI) [1], [2], [3] and the acute respiratory distress syndrome (ARDS) [4], [5]. Following hemorrhagic shock, rats treated with nebulized HTS showed decreased lung leak [6]. HTS directly modulates human neutrophil [7], T-cell [2] and macrophage [8] responses, while HTS resuscitation decreases alveolar macrophage activation and neutrophil recruitment into the lung [1], [2], [8]. However, the effects of HTS on pro-inflammatory activation of lung epithelial cells have not been defined. Tumor necrosis factor-α (TNFα) and interleukin 1β (IL-1β) are pro-inflammatory cytokines [9], [10] whose signaling pathways are expressed ubiquitously in human cells and induce expression of multiple pro-inflammatory cytokines and chemokines. Both TNFα and IL-1β play important roles in pulmonary inflammation and have been implicated in the development of ALI following hemorrhagic shock. Inhibition of these cytokines has been previously shown to diminish lung inflammation [11], [12], [13], [14], [15], [16].

Nuclear factor-κB (NF-κB) represents a potential target for hyperosmolar (HOsm) modulation of inflammation [8], [17]. Dimeric NF-κB, comprised of either p65, p50 or both, plays a central role in the transcription of several genes involved in inflammation in both animals and humans [18], [19], [20], [21]. In animals, hemorrhagic shock induced NF-κB activation in the lung and increased TNFα and IL-1β production [13], [22]. In humans, clinical studies of ARDS have documented increased NF-κB activation [22], [23].

TNFα and IL-1β also activate MAPK modules [9], [10] and converge on the NF-κB regulated transcription, especially activation of the p65 Rel A subunit, by differentially utilizing the canonical IKK-NEMO complex [16]. Although TNFα signaling requires IKKβ associated with NEMO [18], [24], IL-1β signaling is more diverse and can utilize either IKKα or IKKβ (bound to NEMO) resulting in phosphorylation and degradation of I-κBα, which activates NF-κB [18], [24]. Consequently, these stimuli were chosen to contrast and classify potential mechanistic targets of HOsm treatment during receptor-stimulated inflammation in an alveolar pneumocyte line (A549). Additional TNFα and IL-1β-associated, pro-inflammatory transcription factors [25], [26] were also targeted for investigation, including: c-Jun (AP-1 family), STAT-1, JNK, Erk-1/2 and interferon response factor 1 (IRF-1).

To investigate HTS inhibition of ALI and to investigate if the effects were Na^+^ related or true HOsm effects, HTS was compared to a non-ionic, non toxic, clinically readily available substance at similar HOsm: sorbitol. Investigation into HOsm therapy for post-traumatic ALI may elucidate the mechanisms of HTS as a clinical therapy. Therefore we hypothesize that HOsm inhibits TNFα and IL-1β mediated signaling at multiple levels, decreasing cytokine/chemokine production in pulmonary epithelium.

## Materials and Methods

### Cell culture and treatment

A549 pulmonary epithelial cells (CCL-185, ATCC, Manassas, VA, USA) were cultured in Ham's F12 medium with 10% fetal bovine serum and 100 IE/ml penicillin and 0.1 mg/ml streptomycin. Cells were grown to 80–90% confluence for viability measurements (MTT, Cell Proliferation Kit, Roche Biochemicals, Indianapolis IN), cytokine, Western blotting, and phosphoprotein measurement and to 70–80% confluence for indirect immunofluorescent staining and DNA binding assays. A549 were cultured and treated in suspension, with end over end rotation, for cell size measurements by forward scatter (CyAn ADP analyzer,Beckman- Coulter, Indianapolis IN). Hyperosmolar treatments consisted of a 30 minute pre-treatment followed by continued immersion in media supplemented to a final osmolarity of 400±5 mOsm with either sorbitol or NaCl, confirmed by a freezing point depression osmometer (Advanced Instruments, Inc, Norwood, MA, USA). For the study of the effects of osmolarity on cell size, in suspended cells, we performed a dose response to media ranging from iso-osmolar (290 mOsm) up to 600 mOsm with continuous immersion for 18 hours. HOsm concentrations were based upon previously measured concentrations following IV injection of HTS [27]. Cells were treated with isotonic (290 mOsm) medium (controls) or 400 mOsm (HTS, Sor) media, 10 ng/ml of TNFα or 10 ng/ml IL-1β (Sigma-Aldrich, St. Louis, MO, USA).

### Cytokine and phosphoprotein analysis

Cytokine analyses on supernatants were completed with a multiplex bead-based assay (Bio-Rad Laboratories, Hercules, CA, USA) these data were confirmed via sandwich ELISAs (R&D Systems, Minneapolis, MN, USA). Whole cell lysates were generated using M-PER digestion buffer (Thermo Fisher Scientific Inc, Waltham, MA, USA) and were analyzed for cytokine production using identical ELISAs. Protein phosphorylation was measured using a cell-based colorimetric ELISA system which compared phosphorylated (Ser32) to total levels of I-κBα, phosphorylated (Ser73) to total levels of c-Jun and dual phosphorylated to total p38MAPK (SABiosciences, Frederick, MD, USA).

### mRNA Expression

A549 cells grown in 12-well plates were harvested and total RNA extracted using RNeasy Mini Kit (Qiagen Inc., Valencia, CA, USA) according to the manufacturer's instructions. mRNA levels were quantified by two-step real-time PCR. Briefly, 2.5 µg of total RNA per sample was incubated with 3 µg of random primers (Invitrogen Corp., Carslbad, CA, USA) at 70°C for 2 minutes. The reaction was chilled and incubated with reverse transcriptase Superscript II (Invitrogen Corp.) and 1.25 mM dNTP (Applied Biosystems, Foster City, CA, USA) at 42°C for 30 minutes and a third incubation at 70°C. Equal amounts of cDNA were used for the real-time PCR. All reagents, primers and probes are validated Taqman expression assays purchased from Applied Biosystems (RANTES CCL5: Assay ID Hs00174575_m1 cat# 4453320, spans exons 2and 3 with an amplicon length of 63 bp; IP10 CXCL10: Assay ID Hs00171042_m1 cat # 4331182, spans exon 1–2 amplicon length 98 bp; MCP-1 CCL2: Assay ID Hs00234140_m1 Cat. # 433118, spans exon 1–2 amplicon length 99 bp; GAPDH: Assay ID Hs01922876_u1Cat. # 4331182, spans exon 7-7 amplicon length 139 bp). All reactions were performed and analyzed on an iCycler iQ5 multicolor real-time PCR detection system (Bio-Rad Laboratories, Hercules, CA, USA). Levels of RANTES, IP-10 and MCP-1 in each sample were normalized and expressed as fold change over GAPDH.

### Western blot analysis

Protein concentrations were measured using a BCA protein assay (Pierce Biotechnology Inc, Rockford, IL, USA). Nuclear fractions were harvested using a nuclear extract kit and protein quantification kit (40010 Nuclear Extract Kit, Active Motif, Carlsbad, CA, USA). Equal protein concentrations were loaded onto an 8–16% acrylamide gel, fractioned by SDS-PAGE and transferred onto a nitrocellulose membrane (Bio-Rad Laboratories, Hercules, CA, USA) and equal protein loading was further confirmed by 1% Ponceau staining (Sigma-Aldrich, St. Louis, MO, USA). The membranes were blocked for with 5% non-fat milk/PBS solution (1 hour) and probed with antibodies against phosphorylated I-κBα (Ser32/36, Cell Signaling Technology, Danvers, MA, USA), IRF-1 (C-20, Santa Cruz Biotechnology Inc, Santa Cruz, CA, USA), Lamin B (M-20, Santa Cruz Biotechnology Inc)and GAPDH (G9, Santa Cruz Biotechnology Inc, Santa Cruz, CA, USA) at 1 µg/ml, overnight at 4°C, in 5% non-fat milk in PBS/0.05% Tween 20. Anti-species secondary antibodies, at titers (1∶5,000–1∶10,000) (Pierce Biotechnology Inc, Rockford, IL, USA), conjugated to horseradish peroxidase were used for enhanced chemiluminescence detection (ECL) (Pierce Biotechnology Inc). Images were captured and quantified using a ChemiDoc XRS digital chemiluminescent detection system (Bio-Rad Laboratories, Hercules, CA, USA).

### Indirect immunofluorescent stain

A549 cells were grown and treated on glass slides then fixed and permeabilized with 70% acetone/30% methanol solution at −20°C for 10 minutes. The slides were air-dried, blocked with 10% donkey serum in PBS (60 min) (Jackson Immuno Research Laboratories, West Grove, PA, USA), and incubated overnight incubation at 4°C with (1∶50) rabbit polyclonal anti-p65 (NF-κB) antibody in 1% BSA/PBS (Santa-Cruz Biotechnology Inc, Santa Cruz, CA, USA). After washing, the slides were incubated for 1 hour at room temperature with (1∶100) Alexa fluor 488 conjugated donkey anti-rabbit antibody, and Alexa fluor 647 conjugated WGA to stain the membranes ((Molecular Probes Invitrogen Detection Technologies, Eugene, OR, USA). The chromatin stain DAPI was employed to demarcate the nuclei (Sigma-Aldrich, St. Louis, MO, USA). Images were acquired with a Leica DMRXA fitted with a Cooke CCD SensiCam using Chroma Sedat filters with single excitation and emission filter cubes. The three channel images were then digitally processed, for calculations of fluorescent mean intensities and for mean cell size (in voxels) measurements, using Intelligent Imaging Innovations Slidebook 4.1 software (Intelligent Imaging Innovations Inc, Denver, CO, USA).

### Transcription factor DNA binding assay

Activity assays were performed on nuclear fractions of A549 cells and were added at 5 µg of nuclear protein per sample to plates containing transcription factor consensus sequences. Active nuclear forms of the transcription factor studied (NF-κB p65, NF-κB p50 or STAT-1) were detected per the manufacturer's protocol (Active Motif, Carlsbad, CA, USA)

### Statistical Analysis

Data are represented as mean ± standard error of the mean. Comparisons between two groups were assessed by Student's t-test, and those between three or more groups were assessed by analysis of the variance (ANOVA) using JMP 5.0 software (SAS Institute, Inc. Cary, NC USA followed by Bonferreni Dunn correction for multiple comparisons. The comparisons depicted in Table 1 were used to select the candidates for the subsequent specific analysis. The false discovery rate (FDR) was set at 0.15; the q value (corrected level of significance by the Benjamini-Hochberg multiple comparison adjustment method [28]) was calculated and established at <0.018. Variables associated with a significant p-value (p<0.018) by a paired t-test were selected to be further analyzed.

**Table 1:** Hyperosmolarity inhibits secretion of archetypal cytokines induced by TNFα or IL-1β.

Common name	Other name	Chromo-some		*TNF*			*IL-1*	
				+HTS	+Sorb		+HTS	+Sorb
			Fold	*%*	*%*	Fold	*%*	*%*
IL-10	IL10	1q31	-	*-*	*-*			
IL-1b	IL-1B	2q14	1.0	*100.0*	*100.0*			
IL-1ra	IL1F5	2q14.2	1.1	*111.2*	*94.3*	**5.6**	*124.4*	*118.0*
IL-17	IL-17	2q31	-	*-*	*-*	-	*-*	*-*
IL-12	IL12B	3p12	-	*-*	*-*	-	*-*	*-*
IL-8	**CXCL8**	4q12	**136.0**	*163.2*	*169.8*	**400.6**	*146.5*	*138.1*
IP-10	**CXCL10**	4q21	**243.8**	***60.0***	***34.4***	**66.0**	***65.5***	***61.6***
FGF Bas	FGF2	4q26	-	*-*	*-*	-	*-*	*-*
IL-2	IL2	4q26	-	*-*	*-*	-	*-*	*-*
IL-15	IL15	4q31	1.0	*100.0*	*100.0*	2.1	*99.6*	*66.7*
IL-4	IL4	5q31.1	1.1	*95.0*	*95.0*	**6.4**	*70.9*	*82.9*
IL-5	IL5	5q31.1						
IL-9	IL9	5q31.1	2.2	*112.6*	*103.1*	3.1	*87.8*	*83.9*
IL-13	IL13	5q31.1	-	*-*	*-*	-	*-*	*-*
GM-CSF	CSF2	5q31.1	-	*-*	*-*	-	*-*	*-*
TNF-a	TNF-α	6p21.3				3.6	*72.2*	*79.0*
IL-6	IL6	7p21	**58.2**	*123.3*	*106.9*	**406.1**	*75.6*	*91.3*
IL-7	IL7	8q12	-	*-*	*-*	-	*-*	*-*
IFN-g	IFN-γ	12q14	**5.2**	*116.0*	*107.4*	**34.3**	*76.6*	*78.1*
G-CSF	CSF3	17q11.2	1.5	*89.4*	*83.4*	**287.4**	***49.3***	***59.3***
MCP-1	**CCL2**	17q11.2	**830.2**	***47.4***	***52.6***	**550.5**	*97.1*	*95.5*
RANTES	**CCL5**	17q11.2	**113.5**	***9.6***	***4.3***	**426.5**	***11.8***	***9.7***
MIP-1a	**CCL3**	17q11	1.0	*100.0*	*100.0*	1.2	*92.0*	*89.2*
MIP-1b	**CCL4**	17q11	2.0	*53.8*	*50.6*	**16.4**	***49.2***	***47.7***
Eotaxin	**CCL11**	17q21.1	1.0	*100.0*	*100.0*	2.5	*79.4*	*78.4*
PDGF bb		22q13.1	-	*-*	*-*	-	*-*	*-*
VEGF			1.1	*92.1*	*90.0*	2.7	*66.1*	*95.2*

Hyperosmolar effects (400 mOsms, by either NaCl or sorbitol), on 27 inflammation-related gene products secreted by stimulated A549 cells measured by multiplex assay. Chosen from genes clustered on chromosomes 2, 4, 5 and 17 and eight others. Fold increases and inhibition were compiled from three separate experiments. Stimulated responses are bolded, if these measured over 100 ng/ml except for IL-4 (less than 20 ng/ml) **and at least >5 fold**. >400, >100, >5 The effect of HTS and Sorbitol (both 400 mOsm) are shown as *% response* of stimulated values and also underlined if these are at least 33% different, >75, >50, >33. NA, not applicable, – indicates undetectable.

## Results

### Hyperosmolarity inhibits chemokine release and production

To investigate potential anti-inflammatory effects of HOsm on A549 cells, the stimulated production of inflammatory cytokines and chemokines were measured in response to TNFα and IL-1β. Table 1 shows a panel of 27 agents that have been identified in inflammatory lung injury [11], [12], [29], [30], [31], can be measured simultaneously by available multiplexed assay, and sorted by loci. This panel covers the production of various CC and CXC chemokines, growth factors and interleukins that could signal other cells in the pulmonary alveoli such as macrophages, neutrophils and T-cells, disposing toward TH1, 2 or 17 phenotypes. Previous time course experiments demonstrated significant cytokine and chemokine production at 18 hours (data not shown). TNFα induced synthesis and release of six pro-inflammatory cytokines/chemokines, of which three (IP10, MCP-1 and RANTES) were inhibited by HOsm pretreatment. Ten cytokines were stimulated by IL-1β, of which 4 were significantly inhibited by HOsm pretreatment: IP-10, RANTES, G-CSF and MIP-1. Specifically, IL-1β stimulation accounted for greater increases in chemokines and cytokines compared to TNFα except for IP-10 and MCP-1. Moreover, IL-1β stimulation caused increased release of CXCL, which resides on Chr 4, and 3/5 CCL which appear on Chr 17. VEGF was secreted constitutively (about 10 fold over baseline in 18 hrs). HTS and sorbitol were similar in their inhibitory effects on cytokine/chemokine synthesis and release.

**Figure 1:**
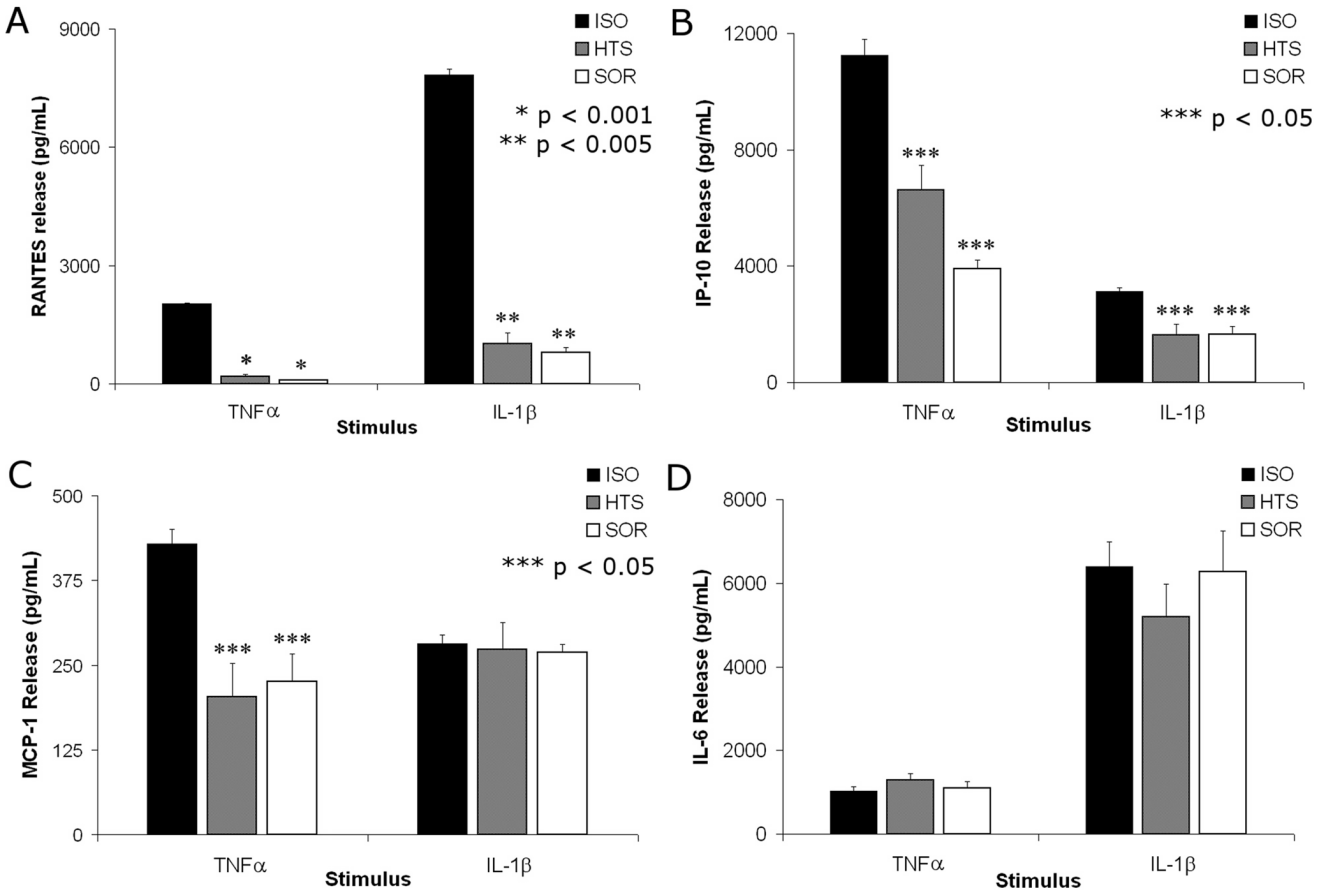
NaCl or sorbitol HOsmolarity (HOsm)inhibit chemokine secretion similarly, depending on stimulus. RANTES (A) and IP-10 (B) release was inhibited by both HTS and SOR when stimulated by TNFα or IL-1β. HTS and SOR inhibited MCP-1 (C) release only when stimulated by TNFα but not by IL-1β. HOsm did not inhibit IL-6 (D) release with either stimulus. No significant differences were seen between HTS and SOR groups. Chemokine production was measured at 18 hours when treated with TNFα or IL-1β in the presence of 400 mOsm HTS or Sorbitol.

ELISAs confirmed the four observed patterns of response to TNFα and IL-1β (Fig. 1). The inhibitory effects of both HTS and SOR were similar on TNFα and IL-1β stimulated increases in RANTES/CCL5 concentration, with 90-95% inhibition compared to isotonic media, p<0.005 [Fig. 1a]. The 40–65% inhibition of IP-10/CXCL10 was proportionate for both stimuli despite different levels of expression (p<0.05) [Figure 1b]. TNFα-induced MCP-1/CCL2 release was inhibited 40–50% by HOsm (p<0.05) [Figure 1c], but IL-1β-induced MCP-1release remained was unaffected. IL-6 synthesis and release was induced by both stimuli and unaffected by HOsm [Figure 1d]. IL-1β stimulation resulted in production of MIP-1β/CCL4 and G-CSF/CSF 3, which were inhibited by HOsm. Furthermore HOsm inhibited both IL-1β-induced IFN-γ (but not TNFα) and TNFα production. Both stimuli increased production of IL-6, VEGF, IL-8, and IL-9 and were not affected by HOsm [Table 1]. No significant differences were noted between HTS and SOR treatments; thus, HOsm appears to inhibit production of multiple cytokines/chemokines dependent upon the stimulus employed. Lastly, because decreased cytokine levels in the supernatant could be due to inhibition of release rather than inhibition of synthesis or increase in degradation, ELISAs for RANTES were performed on whole cell lysates. Total cellular RANTES) concentrations (TABLE 2) demonstrated a similar pattern as the supernatants, indicating the observed inhibition is likely due to either to decreased synthesis or to increased degradation.

**Table 2:** HOsm treatment inhibits intracellular RANTES levels in parallel with overall chemokine release levels.

	TNFα	TNFα + HTS	TNFα + Sor	IL-1β	IL-1β + HTS	IL-1β + Sor
Mean (pg/ml)	610±48	108±73	48±14	252±33	37±8	39±4
p value		0.05	0.01		0.01	0.01

Whole cell lysates were analyzed by ELISA at 18 hours after treatment with TNFα or IL-1β in the presence of 400 mOsm HTS or Sorbitol. p<0.05 and p<0.01 for differences between ISO and HTS or SOR groups; no significant differences were seen between HTS and SOR groups.

### Hyperosmolar treatment affects mRNA translation

**Figure 2:**
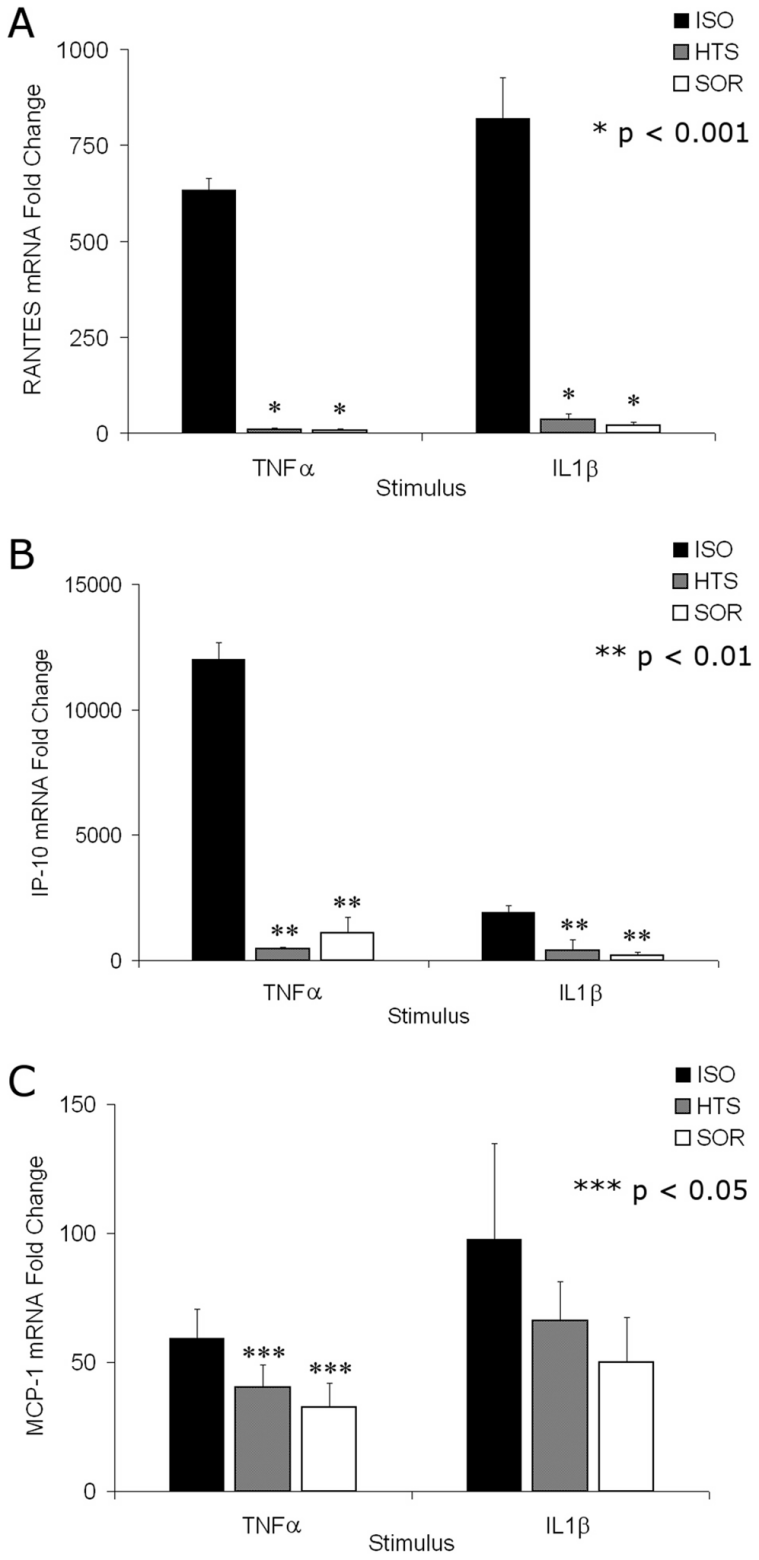
HOsm inhibits TNFα or IL-1β stimulated mRNA for RANTES (A), IP-10 (B) and MCP-1 (C) chemokines in parallel with overall chemokine release levels. mRNA levels were measured following stimulation for 6 hours and normalized to constitutive GAPDH production after treatment with TNFα or IL-1β in the presence of 400 mOsm HTS or Sorbitol. No significant differences were seen between any of the HTS and SOR groups.

To determine if HOsm inhibition was due to decrease in mRNA expression, the intracellular mRNA levels of RANTES, IP-10, and MCP-1 were measured. These mRNA levels matched the released concentrations of these chemokines into the supernatant for both TNFα and IL-1β indicating that the inhibition may be upstream of mRNA [Figure 2].

### Hyperosmolarity decreased p65 translocation with TNFα but not IL-1β stimulation

**Figure 3:**
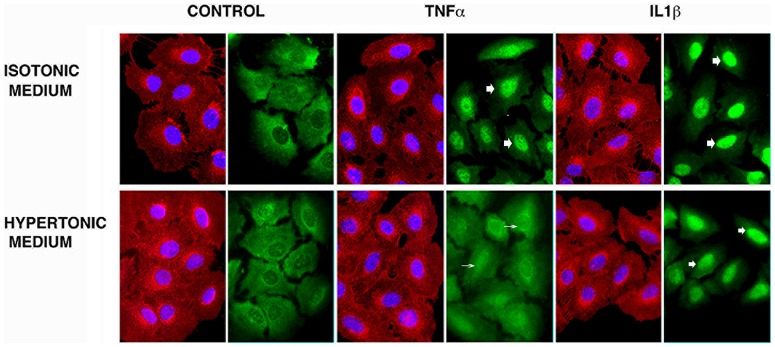
HOsm inhibits translocation of NF-κB into the nucleus induced by TNFα stimulation but not by IL-1β stimulation. Indirect immunofluorescent staining was used to examine the intracellular location of the p65 subunit of NF-κB following 30 minutes of TNFα or IL-1β stimulation in the presence of isotonic (upper panels) or 400 mOsm HTS (SOR group not shown but appeared similar to HTS group). Cell structure is depicted in red (WGA glycoprotein stain), nuclei in blue (DAPI) and NF-κB in green. NF-kB translocation into the nucleus is seen in TNFα, IL-1β treated cells in isotonic medium. Under hyperosmolar conditions, translocation (thick arrows) stimulated by TNFα (but not IL-1β) is diminished (thin arrows). This figure is representative of three separate experiments. The bottom panel shows that **HOsm does not significantly affect cell viability.** Viabilty of pulmonary epithelial cells measured by MTT assay (absorbance) at 18 hours in the presence of 400 mOsm HTS or SOR. Cells were killed by heat treatment, at 90°Cfor 30 minutes prior to assay.

HOsm treatment decreased p65 sub-unit of NF-κB translocation into the nucleus following TNFα but not IL-1β stimulation [Figure 3].

### Hyperosmolarity decreased NF-κB activation with TNFα but not IL-1β stimulation

**Figure 4:**
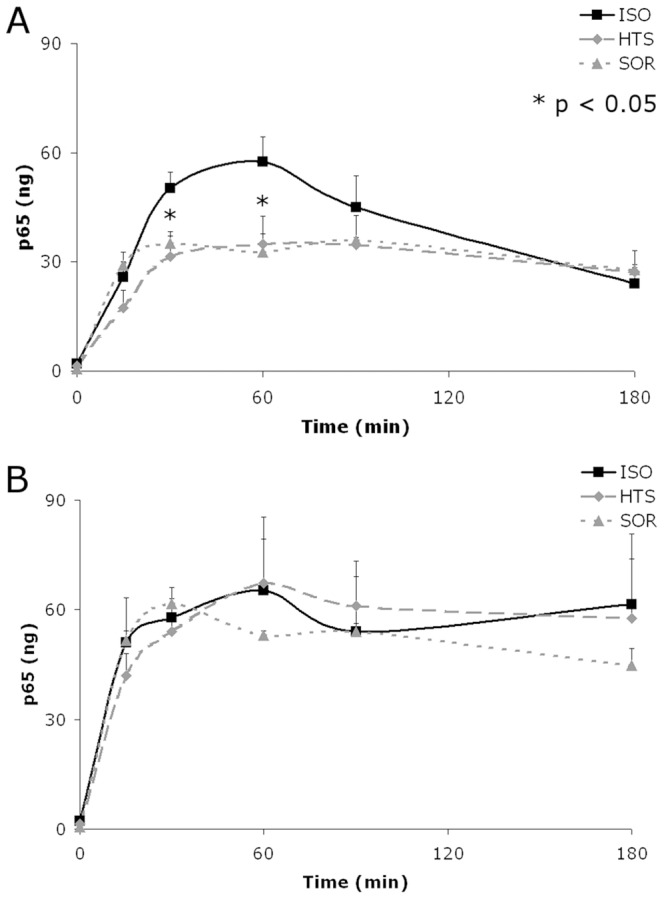
HOsm inhibits NF-κB activation by TNFα but not IL-1β stimulation. The time course of NF-κB consensus binding of the p65 subunit in nuclear fraction of cells stimulated by TNFα (A) or IL-1β (B) in the presence of 400 mOsm HTS or SOR (n = 5). Activation assayed by DNA binding to consensus sequence; * p<0.05 between ISO and HTS or SOR groups at matched time points; no significant differences were found between HTS and SOR groups.

To quantify and further explore the effects of HOsm on NF-κB, a DNA binding assay to examine the activity of nuclear NF-κB was employed and, as an initial required preliminary, the effects of TNFα and IL-1β on NF-κB with respect to its functional differences in nuclear binding activity. NF-κB activation elicits a heterodimer between the p65 and p50 sub-units of NF-κB, according to the canonical pathway [20], [32], [33], and the described experiments focused on the p65 sub-unit, although samples run with the p50 sub-unit showed results similar to that of p65 (data not shown). At both the 30 and 60 minutes HOsm produced significant inhibition of TNFα-stimulated NF-κB activation (p<.05) [Fig 4A]; however, IL-1β produced no significant differences at any of the time points studied [Fig 4B]. TNFα stimulation of pulmonary epithelial cells in isotonic media produced an NF-κB peak activation equivalent to 60 ng of purified p65 before diminishing to a level of 30 ng, equivalent to the peak and sustained levels produced by HOsm treated cells. In contrast, IL-1β stimulated cells maintained an activation of around 60 ng across the entire time course, independent of HOsm treatment.

### Hyperosmolar treatment decreases I-κBα phosphorylation

**Figure 5:**
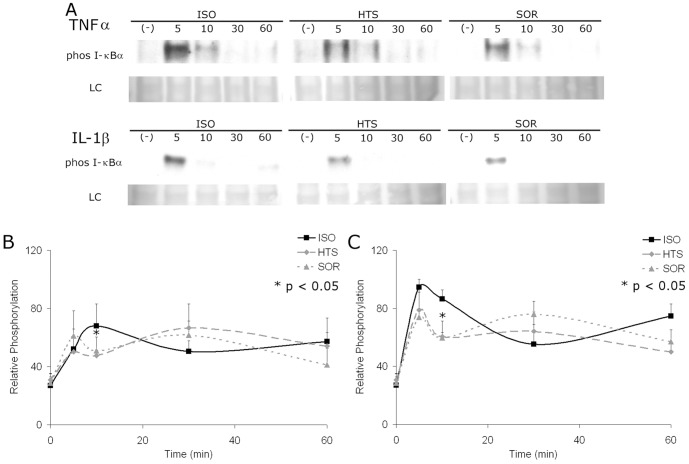
HOsm inhibits phosphorylation of I-κBα induced by both TNFα and IL-1β. Phosphorylation of I-κBα is inhibited at 10 minutes in cells stimulated with TNFα or IL-1β (10 ng/ml) by either 400 mOsm HTS or SOR treatment. The immunoblot is representative of five separate experiments. LC represent the loading control obtained by Ponceau-S stain of the nitro cellulose membranes. Phosphoprotein ELISA time course of cells stimulated by TNFα (B) or IL-1β (C) (both 10 ng/ml), in the presence of 400 mOsm HTS or Sorbitol; normalized for phosphoprotein/total protein levels. Phosphorylation of I-κBα initially diminished with either TNFα (B) or IL-1β (C) stimulation when treated with HOsm; however, I-κBα phosphorylation increases at 30 minutes with HOsm over isotonic treatment with either stimulus; * p<0.05 for ISO vs. either HTS or SOR at matched time points; no significant differences were found between HTS and SOR groups.

I-κBα phosphorylation represents a key regulatory step in NF-κB activation, leading to release of I-κBα from its inhibitory role on NF-κB. Western blot analysis of I-κBα with both stimuli in the presence of HOsm demonstrated a decrease in phosphorylation [Fig 5]. This inhibition was noted most strongly at 5 minutes after stimulation. When quantified using a phosphoprotein ELISA, inhibition of I-κBα phosphorylation was noted at 10 minutes following stimulation with either TNFα or IL-1β (p<0.05) [Figure 5B&C]. Interestingly, with either TNFα or IL-1β stimulation, I-κBα phosphorylation in the setting of HOsm treatment peaked at 30 minutes at or above the levels seen in isotonic media at the same time point. Thus, HOsm diminishes I-κBα phosphorylation similarly with either TNFα or IL-1β stimulation at the early time points but appears to only delay peak phosphorylation.

### Transcription factor IRF-1 nuclear localization was inhibited by HOsm with both TNFα and IL-1β stimulation

**Figure 6:**
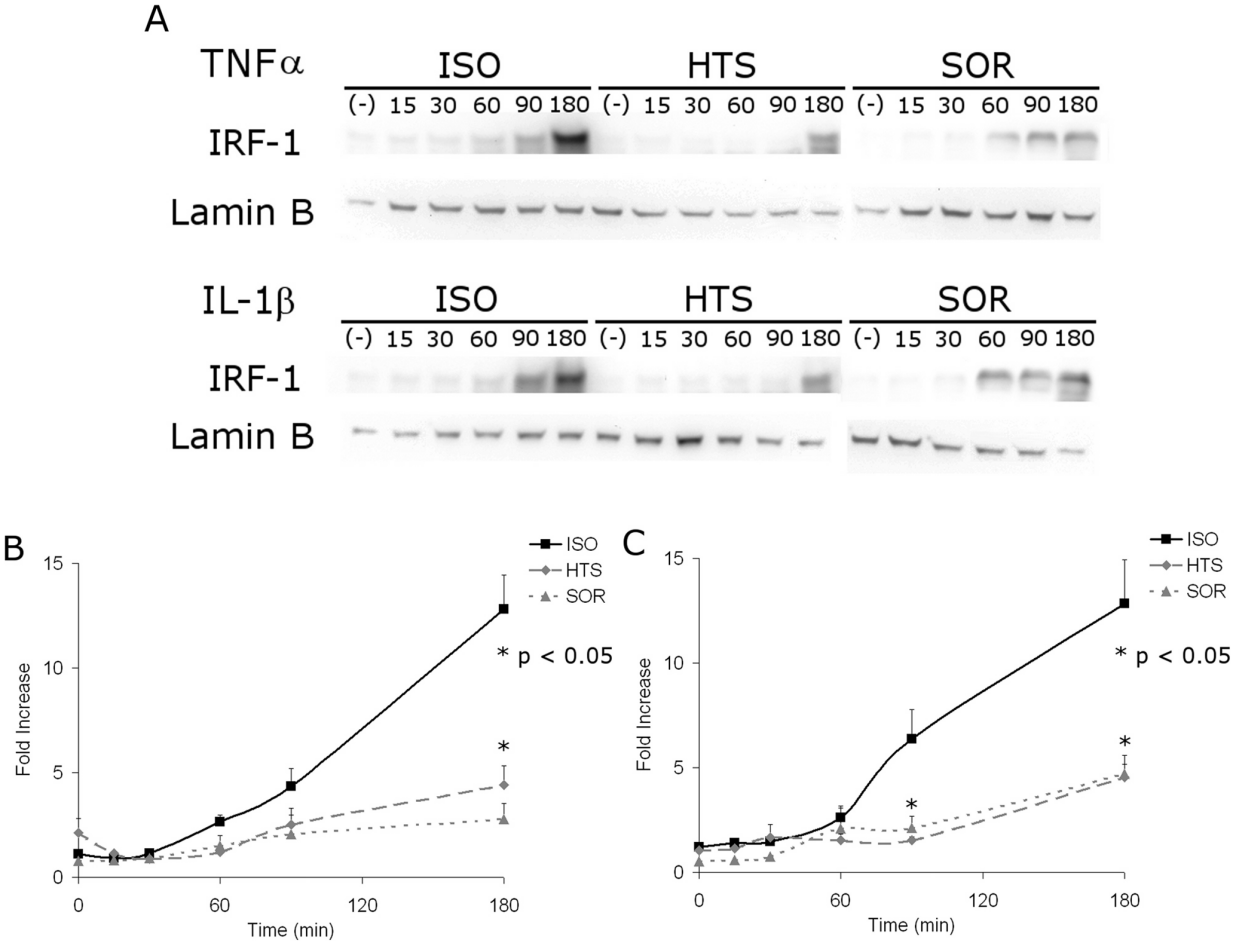
HOsm inhibits IRF-1 localization to the nucleus with either TNFα or IL-1β stimulation. IRF-1 in the nuclear extract of cells stimulated by TNFα or IL-1β in the presence of 400 mOsm HTS or sorbitol was examined by immunoblotting (A), figure is representative of three separate experiments. The gels were digitized and quantitated for nuclear IRF-1 after TNFα (A) or IL-1β stimulation (C); * p<0.05 for ISO vs. either HTS or SOR at matched time points; no significant differences were found between HTS and SOR groups.

Based on the RANTES promoter region [26], [34], IRF-1 was identified as a proximal transcription factor and the increases in TNFα- or IL-1β-induced in nuclear IRF-1 levels were analyzed by western blotting [Figure 6A]. In these experiments the IRF-1 levels on western blots were quantified by densitomery and IRF-1 levels were calculated as a fold change from nuclear IRF-1 densitometry from control cells. TNFα- and IL-1β- mediated increases in nuclear IRF-1 levels in A549 cells were inhibited by HOsm at 180 minutes [Figure 6B] and at 90 and 180 minutes [Figure 6C], respectively (p<0.05). The HTS and SOR groups were not statistically different.

## Discussion

Of the pro-inflammatory mediators investigated in the preliminary screen, TNFα apparently increased the release of six cytokines/chemokines (>5-fold), of which 3 were inhibited by HOsm pretreatment: RANTES, MCP-1 and IP-10. In contrast, IL-1β induced 10 cytokines/chemokines of which HOsm attenuated 4: RANTES, IP-10, G-CSF and MIP1β. The 6 mediators that seemed increased in response to TNFα were also induced by IL-1β; but, IP-10 and MCP-1 were more responsive to TNFα. Thus, IL-1β apparently induced release of a larger array of cytokines/chemokines than TNFα, at identical concentrations, suggesting different modes of transcriptional activation.

**Figure 7:**
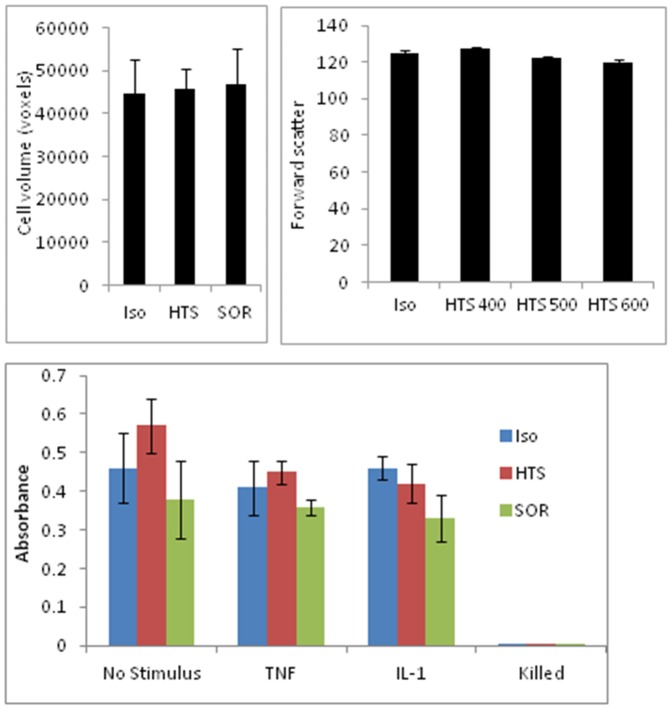
HOsm does not significantly affect cell volume. Top left panel shows the mean total cell volume of pulmonary epithelial cells measured by immunofluorescent microscopy is not changed in the presence of 400 mOsm HTS or SOR after 18 hours of incubation (n = 3). The panel on top right shows mean (± sem) forward scatter of pulmonary epithelial cells measured by flow cytometry is also unchanged in the presence of isotonic and 400, 500, and 600 mOsm solutions of media plus HTS, after 18 hours of incubation (n = 3).

Hyperosmolarity represents a promising potential therapy against inflammation. Both NaCl and sorbitol can be used to prepare hyperosmolar solutions and are clinically accessible. While NaCl is ionized in aqueous solutions, sorbitol is a C6 polyol (an open chain configuration of an hexose). We used these to determine whether the intracellular response to HOsm required ion flux to achieve anti-inflammatory benefit. We and others have used modest hyperosmolarity of about 400 mOsm against various cells [1], [27], [35], [36], [37]. These doses were derived from peak levels achievable in animal models and substantial cell shrinkage or death (Fig 7) are not evident. We have also used inhaled aerosolized hypertonic saline to protect animal lungs but the local dose is unknown [6].

The mechanism by which modest HOsm (∼400 mOsm) reduces inflammatory signaling is unclear [1], [6], [35], [37]. The cellular response to hyperosmolarity (HOsm) is an ancient pathway that led to the discovery of the modular p38MAPK (originally called hyperosmolar glycerol 1 HOG1 in yeast) pathways [38]. Some have suggested ionic mechanisms, others invoked cytostructural elements, but we have not found evidence for these in A549 and other mammalian cells [17], [36].

Hyperosmolarity inhibited RANTES and IP-10 production, which requires NF-κB, whether induced by TNFα or IL-1β. Production of the NF-κB-dependent chemokine MCP-1 was inhibited by HOsm in response to TNFα but not IL-1β. But G-CSF and MIP-1β, both are NF-κB dependent, could only be induced by IL-1β, but not TNFα, and appeared inhibited by HOsm. Irrespective of the pro-inflammatory stimulus, A549 cells demonstrated similar HOsm inhibition, suggesting a similar non-ionic mechanism. In contrast, production of the ubiquitous, pro-inflammatory agents IL-6, IFNγ and IL-8 appeared increased by both TNFα and IL-1β but not inhibited by HOsm. Similar to previous results [37], [39], but here at more modest HOsm, TNFα- and IL-1β-elicited release of the neutrophil chemoattractant IL-8 was increased (see also below). The reasons for this increase are unclear, but again argues against cell dysfunction.

Due to their central role in inflammation, TNFα and IL-1β signal transduction pathways have been well delineated [9], [10], [40], [41]. Following ligand binding, both receptors recruit distinct intracellular proteins that converge on the I-κB kinase (IKK) complex activating the NF-κB pathway [18], [40], [41]. NF-κB is a dimer held in the cytoplasm by its inhibitor, I-κBα, which masks its nuclear localization sequence. Upon stimulation through receptor signaling pathways (including TNFR, IL-1R or TLR) the IKK complex phosphorylates I-κBα at serine residues 32/36, leading to I-κBα degradation by the proteosome pathway. The NF-κB dimer translocates into the nucleus and begins transcription of many pro-inflammatory genes [40], [41]. The TNFα and IL-1β pathways are also known to selectively activate other signal transduction processes, such as various MAPK modules [9], [10], [42], [43] which could independently affect pro-inflammatory chemokine production.

Because multiple transcription factors have been implicated in pro-inflammatory activation of various cells [25], [26] a number of potential transcription factors implicated in promoting cytokines that were inhibited by HOsm were investigated (Table 1). Firstly, HOsm did not affect TNFα- or IL-1β-elicited phosphorylation of Erk-1/2, JNK, or of c-Jun (a key component of AP-1) at 30 or 60 minutes by phosphoprotein analysis (data not shown). STAT-1 activation, as measured by a DNA binding assay, was also tested but no detectable STAT-1 binding was found in the nuclear fractions of any treatment groups (data not shown). Lastly, HOsm did not inhibit TNFα- or IL-1β-induced p38MAPK dual phosphorylation (data not shown).

IL-1β caused increased NF-κB nuclear translocation for both the p65 and p50 subunits of the dimer, which is an expected consequence of greater I-κBα activation in the first 30 minutes, similar to previous data, which demonstrated that either IKKα or IKKβ were capable of phosphorylating I-κBα [18]. In contrast, TNFα requires both IKKα and IKKβ for I-κBα phosphorylation. The phosphorylation of c-Jun and nuclear translocation of IRF-1 seem comparable with either stimulus; Erk-1/2, JNK, and STAT-1 do not seem to play a role in the A549 cellular response to HOsm inhibition of pro-inflammatory stimuli, contrary to their roles in other cell types [42], [44], [45]. Previously, in human vascular smooth muscle cells, we found that both TNFα and HOsm induced p38MAPK activation, additively [17]. Here, inhibiting p38MAPK reduced proinflammatory markers but not as much as HTS. Thus the mechanisms of HOsm inhibition is probably unrelated to p38MAPK [17], [35].

At first glance, the early attenuation of TNFα-stimulated NF-κB translocation to the nucleus after HOsm pretreatment offers a satisfactory explanation for the attenuated expression of early NF-κB-driven genes such as RANTES or MCP-1. However, HOsm does not affect IL1β-driven NF-κB translocation, while suppressing cytokine expression. These data indicate that NF-κB promoter activity may be insufficient to explain the inhibition of cytokine/chemokine expression. To assess other HOsm suppression mechanisms, other TFs regulating RANTES-type promoter modules were examined [26]. After either stimulus, nuclear IRF-1 increased over 3 hours. Nuclear fractions showed that IRF-1 levels were elevated by 60 minutes and significantly reduced as early as 90 minutes for both. This decrease implies that HOsm may attenuate gene transcription by retarding the nuclear translocation of several, transcription factors.

Analysis of lavage fluids and injured lungs from animal studies and human clinical trials have found elevated levels of a variety of CXC (IL-8, IP-10) and CC chemokines (RANTES, MCP-1, MIP), which often correlate with leukocyte infiltration and outcomes [11], [46], [47]. Many of these (e.g. IL-8, RANTES/CCL5, MCP-1/CCL2, MIP1β/CCL4) are produced by alveolar Type II cells as part of their role in lung defense [48]. Although many of these gene products require NF-κB for expression, the actual promoter elements differ in their affinity [49]. These promoter regions also contain other transcription factor module binding elements such as for AP-1, C/EBP and IRF which interact with NF-κB to further regulate transcription [26], [49].

The promoter region of RANTES has specifically been examined with bioinformatics [26]. In several cell types, the NF-κB promoter region of RANTES is neighbored by sites for other promoter modules, including IRF-1. However, there was no inhibition of c-jun phosphorylation (a component of AP-1) nor of STAT-1 DNA-binding (data not shown). This study in lung epithelial cells recapitulates previous work in endothelial cells stimulated by TNFα and IL-1β, where hyperosmolarity inhibited VCAM-1 production [50] via decreased IRF-1 activation. Further bioinformatics and TF translocation details are required to explain the selective stimulation and blockade of G-CSF and MIP-1β with IL-1β but not TNFα. Moreover, why does HOsm block MCP-1 with TNFα but not IL-1β? Lastly, the 25-30% suppression by HOsm of IL-6 IL-4 and IFNγ when stimulated by IL-1β may deserve attention. The answers may involve the effects of HOsm on the IKK signalosome and gene specific transcription assembly.

While promoter organizations of IP-10, IL-6 and IL-1β genes resemble that of RANTES in some cell types [26], HOsm inhibited IP-10 production but did not suppress IL-6 release nor induce IL-1β production by TNFα. The striking lack of inhibition of IL-8 (a well-known NF-κB-regulated gene) by HOsm to either stimulus may be due to the tight binding of NF-κB to the **consensus** IL-8 promoter site [49], making this association impervious to the about 50% decrease in nuclear NF-kb levels.(Fig 4). The related, but weaker, NF-κB binding site in MCP-1 does not appear to contain a neighboring IRF-1 binding module, which may explain why HOsm treatment inhibits production of MCP-1 only with TNFα but not IL-1β (Table 1).

This study demonstrates that HOsm selectively inhibits the production of some pro-inflammatory mediators but not others; however, the synthesis and release of some specific agents involved in pulmonary epithelial inflammation (RANTES, IP-10, G-CSF, MIP-1β) are inhibited by HOsm. In A549 cells, HOsm produces effects on nuclear NF-κB, depending upon the stimulus. Controversy exists over the mechanism by which HTS acts in CF; clinical improvement may be due to mucolytic action, airway hydration or anti-inflammatory effects [51], [52]. The effects of HOsm on inflammatory cell signaling could relate to the beneficial application of inhaled HTS in cystic fibrosis patients; furthermore, HTS inhalation may treat or prevent inflammation and/or ALI/ARDS following trauma in a broader patient population.

In summary, HOsm alters the TNFα- and IL-1β signaling in pulmonary epithelial cells thereby reducing the production of selected pro-inflammatory cytokines. The concordance between HTS and sorbitol suggests that the inhibitory mechanisms depend on hyperosmolarity and appear multi-pronged, affecting TNFα and IL-1β signaling cascades at distinct steps. Both HOsm conditions inhibit I-κBα degradation and p65 translocation when TNFα is stimulus, but not with IL-1β. The inhibition of IL-1β reveals another mechanism involving inhibition of IRF-1. IRF-1 is inhibited by both HOsm conditions for both stimuli. A broader analysis of how hyperosmolarity regulates transcription promoters of chemokine gene expression in different cellular contexts should lead to a better understanding of the anti-inflammatory properties of HOsm.
